# Prevalence of Choroidal Abnormalities and Lisch Nodules in Children Meeting Clinical and Molecular Diagnosis of Neurofibromatosis Type 1

**DOI:** 10.1167/tvst.11.2.10

**Published:** 2022-02-04

**Authors:** Mariana Flores Pimentel, Anna Heath, Michael J. Wan, Rowaida Hussein, Kate E. Leahy, Heather MacDonald, Erika Tavares, Cynthia VandenHoven, Katelyn MacNeill, Peter Kannu, Patricia C. Parkin, Elise Heon, Arun Reginald, Ajoy Vincent

**Affiliations:** 1 Department of Ophthalmology and Visual Sciences, The Hospital for Sick Children, Toronto, Ontario, Canada; 2 Department of Ophthalmology and Vision Science, University of Toronto, Toronto, Ontario, Canada; 3 Division of Biostatistics, Dalla Lana School of Public Health, University of Toronto, Ontario, Canada; 4 Genetics & Genome Biology, The Hospital for Sick Children, Toronto, Ontario, Canada; 5 Department of Molecular Genetics, University of Toronto, Toronto, Ontario, Canada; 6 Department of Genetic Counselling, The Hospital for Sick Children, Toronto, Ontario, Canada; 7 Department of Medical Genetics, University of Alberta, Edmonton, Alberta, Canada; 8 Department of Pediatrics, The Hospital for Sick Children, Toronto, Ontario, Canada

**Keywords:** NF1, neurofibromatosis, neurofibromin, genetics, near-infrared imaging, eye diseases, hereditary, choroidal diseases

## Abstract

**Purpose:**

To determine the prevalence of choroidal abnormalities (CAs) and Lisch nodules (LNs) in children who met the clinical diagnostic criteria (CDC) alone and those with a molecularly confirmed diagnosis (MCD) of neurofibromatosis type 1 (NF1), and to ascertain any differences between the groups.

**Methods:**

This was a cross-sectional observational study. All children who met the CDC and/or had MCD of NF1 and underwent eye examination were included. At least two CAs or LNs between the two eyes were set as a threshold to define the presence of either abnormality. Frequencies alongside 95% confidence intervals (CIs) were calculated. The relationship between patient age and the presence of LNs and/or CAs was estimated using logistic regression.

**Results:**

The study cohort included 94 patients; CAs (64%) were more prevalent than LNs (41%) (0.22; 95% CI, 0.08–0.36; *P* = 0.0023). The probability of the presence of LNs was lower than that of CAs across all ages (odds ratio = 0.37; 95% CI, 0.20–0.69; *P* = 0.00173). CAs were exclusively found in 37% of patients and LNs in 16%; 80% had either CAs or LNs, or both. In the CDC group (*n* = 41), the difference in prevalence (CAs = 68%, LNs = 51%) did not attain statistical significance (0.17; 95% CI, −0.06 to 0.40; *P* = 0.18). In the MCD group (*n* = 53), the difference in prevalence (CAs = 60%, LNs = 34%) was significant (0.26; 95% CI, 0.006–0.47; *P* = 0.023).

**Conclusions:**

CAs were more frequent than LNs in pediatric NF1 patients regardless of age and MCD status. Combining ophthalmological exams with near-infrared imaging will increase the diagnostic reach in pediatric NF1.

**Translational Relevance:**

CAs detected on near-infrared imaging are objective biomarkers in NF1. They are more prevalent and detected earlier in the pediatric population compared with LNs. Hence, the presence of CAs should be routinely ascertained in children suspected with NF1.

## Introduction

Neurofibromatosis type 1 (NF1) is an autosomal dominant multisystem disorder with tumor predisposition caused by heterozygous pathogenic variants in the neurofibromin gene (*NF1*, located on chromosome 17q11.2), which encodes for a tumor suppressor protein.^1^ Penetrance is virtually 100%, but expression is highly variable. When the National Institutes of Health (NIH) established the NF1 clinical diagnostic criteria (CDC) in 1987,^2^ they were largely based on underlying involvement of the skin, bone, and nervous system. The presence of two or more of the following criteria were required for diagnosis: six or more café-au-lait macules (CALMs), axillary or inguinal freckling, two or more cutaneous neurofibromas or one plexiform neurofibroma, distinctive osseous lesions (pseudarthrosis, sphenoid wing hypoplasia), optic glioma, two or more iris Lisch nodules, and a first-degree affected relative.^2^^,^^3^ The identification of overlapping phenotypes has since fueled research on diagnostic classification, and, in the recently published NF1 International Revised Consensus,^4^ a recommendation was made to modify an existing criterion—from “first-degree relative with NF1” to “a parent with NF1”—while adding two new criteria: choroidal abnormalities observed on near-infrared (NIR) imaging and the presence of a heterozygous pathogenic *NF1* variant with a variant allele fraction of 50% in apparently normal tissue.^4^ Further, in the 2021 recommendation, the presence of either two CAs or LNs was assigned a diagnostic criteria point for NF1.^4^

CALMs are seen in virtually every patient with NF1 (frequency >99%) by 12 years of age, with the majority (96.7%) manifesting more than six macules by 3 years of age.^5^ The number and size of CALMs are known to increase over the first 5 to 7 years of life.^6^ Ocular manifestations can be present in early childhood (e.g., optic pathway glioma, with a prevalence of up to 15% but only 5% symptomatic)^3^ or manifest in later childhood and adolescence as Lisch nodules (LNs). The earliest onset of LNs (raised tan-colored iris hamartomas) is thought to be around 3 years, and their prevalence increases with age. In children, LNs are reported in 43% of those < 12 years old^7^ and 57% to 75% of those ≤ 15 years old^8^^,^^9^; LNs are reported in >90% of adults with NF1.^3^ Similarly, the prevalence of all clinical manifestations of NF1 are variable and may be age related.^3^

Choroidal neurofibromatosis was first characterized on histopathology by Wolter et al.^10^ in 1962 as ovoid bodies of proliferating neoplastic Schwann cells arranged in concentric rings around axons. Yasunari et al.^11^ were the first to image choroidal neurofibromatosis in vivo, and they described it as bright patchy regions on infrared fundus examination. NIR imaging is being increasingly used to identify these choroidal abnormalities (CAs), most commonly, in the posterior pole.^7^^,^^8^ Despite a lack of histopathological correlation, it is presumed that choroidal neurofibromatosis is visualized as CAs on NIR imaging, possibly due to (1) choroidal thickening and (2) an increase in number of melanocytes in the area.^8^^,^^12^^,^^13^ Further, two subtypes of CAs have been described: (1) rounded, bright, well-defined, and easily identifiable, and (2) patchy, dull, irregular, and poorly defined.^14^ On spectral-domain optical coherence tomography, these CAs show as hyperreflective dome-shaped or hyperreflective placoid formations, respectively.^14^

In adult patient cohorts meeting the CDC for NF1, the reported prevalence of CAs and that of LNs were similar, ranging from 82% to 100% and 82% to 90%, respectively.^7^^,^^11^^,^^13^ In pediatric cohorts that meet the CDC, the prevalence of CAs (60%–100%) is presumed to be higher than that of LNs (57%–75%).^7^^–^^9^^,^^11^^,^^15^ Further, inter-observer agreement for examinations is higher for CAs than LNs (κ = 0.857 for CAs^7^ vs. κ = 0.1774 for LNs^16^). In addition, CAs may be present earlier in life than LNs^8^^,^^13^^,^^17^; if true, then using NIR imaging to assess for the presence of CAs^7^^,^^18^^–^^21^ could accelerate diagnosis even when LNs are absent.

Offering molecular testing to confirm the clinical diagnosis has become the standard of care in Mendelian disorders.^3^ To the authors’ knowledge, the prevalence of CAs and LNs in children with a molecularly confirmed diagnosis (MCD) of NF1 is yet to be determined. The primary aim of the study was to determine the prevalence of CAs and LNs in a cohort of children that met the CDC for NF1 and those with MCD and to identify any difference between the groups. The secondary aims of the study included (1) reporting the probability of finding CAs or LNs across all ages in the entire cohort; (2) reporting the probability of solely finding CAs or LNs across the cohort; (3) ascertaining the prevalence of these clinical ocular signs in the cohort in accordance with the latest diagnostic criteria^4^; and (4) reporting the genetic variants identified and evaluate for any relationship of the class of genetic variant to the prevalence of CAs or LNs.

## Materials and Methods

This was a retrospective cross-sectional observational study. The study protocol was approved by the Institutional Research Ethics Board at the Hospital for Sick Children, Toronto, and conducted in accordance with the tenets of the Declaration of Helsinki. All pediatric patients (≤18 years of age) were eligible if they met the CDC^2^^,^^3^ and/or had a MCD of NF1 and had undergone eye examination and NIR imaging at the Hospital of Sick Children between January 2001 and February 2020. Data were collected from electronic health records (demographic information, family history, and details of eye examination) and ancillary imaging. In patients who were routinely followed, information from the first visit with complete data was used for the analysis.

The patients were divided in two subgroups. Group A included patients who met the CDC (according to the NIH criteria^2^^,^^3^ without genetic testing), and group B included those who, in addition to meeting the CDC, had a MCD, defined as those carrying a pathogenic or likely pathogenic variant in *NF1* identified in a Clinical Laboratory Improvement Amendments–certified laboratory. Best-corrected distance visual acuity (BCVA) was collected from each eye and converted to logarithm of the minimum angle of resolution (logMAR). Patients who could only count fingers, had light perception, or had no light perception were assigned logMAR values of 2.6, 2.8, and 2.9, respectively.^22^ The average BCVA of the two eyes was used for analysis. Documentation of iris LNs was obtained from slit-lamp examinations, external photographs, or clinic notes. To quantify LNs, we used the scale described by Makino et al.^23^ (summed number of iris nodules found among the two eyes). In this study, the presence of two or more LNs, or Makino II, was considered to be the cutoff for the presence of LNs.

The CAs were defined on NIR imaging as (1) rounded and well-defined; (2) patchy, dull, and poorly defined; or (3) a combination of both. NIR imaging was performed with either the Cirrus 4000 (820-nm wavelength; Carl Zeiss Meditec, Jena, Germany) or the SPECTRALIS OCT (815-nm wavelength; Heidelberg Engineering, Heidelberg, Germany). The presence of CAs was determined by counting the number of these lesions in an area within the 30° (and 55° when available) of the retina centered on the fovea following mydriasis. In the literature, the cutoff for clinical significance has been described as 1.5 CAs, between the two eyes.^7^ In this study, we considered the presence of at least two hyperreflective choroidal spots between the two eyes as the threshold to define the presence of CAs. All of the images were analyzed in a blinded manner by two independent investigators (M.F.P., A.V.) to improve accuracy.

### Statistical Analysis

Data were analyzed for the entire cohort and then separately for those patients who met the CDC alone (group A) and those with MCD (group B). Demographic characteristics, age, and BCVA were summarized using descriptive statistics. All statistical analyses were undertaken using the R Language for Statistical Computing.^24^ The proportions of patients with CAs and LNs were calculated and are reported alongside 95% confidence intervals (CIs). To determine if the proportion of patients with CAs was higher than the proportion of patients with LNs, we used a paired *t*-test to compute the risk differences, adjusting for CAs and LNs being measured in the same patients. Logistic regression was used to explore the relationship between the patients’ age and the presence of CAs and/or LNs in the different diagnosis groups and plotted with 95% predictive intervals.^25^ *P* values of 0.05 or less were considered statistically significant. Finally, Benjamini–Hochberg correction was used to control the family-wide error rate across the secondary and subgroup analyses.^26^

## Results

### Entire Cohort

A total of 94 patients met the study criteria and included 56 females (60%) and 38 males (40%). Ages ranged from 3 to 18 years (median, 10 years; mean ± SD, 10.25 ± 4.15 years). The mean BCVA in the cohort was 0.21 ± 0.36 logMAR (range, 0–2.8). Seventy-nine patients (82%) had a BCVA ≥ 0.3 logMAR (or 20/40). All 15 patients with BCVA worse than 0.3 logMAR had coexisting optic pathway glioma.

**Figure 1.**
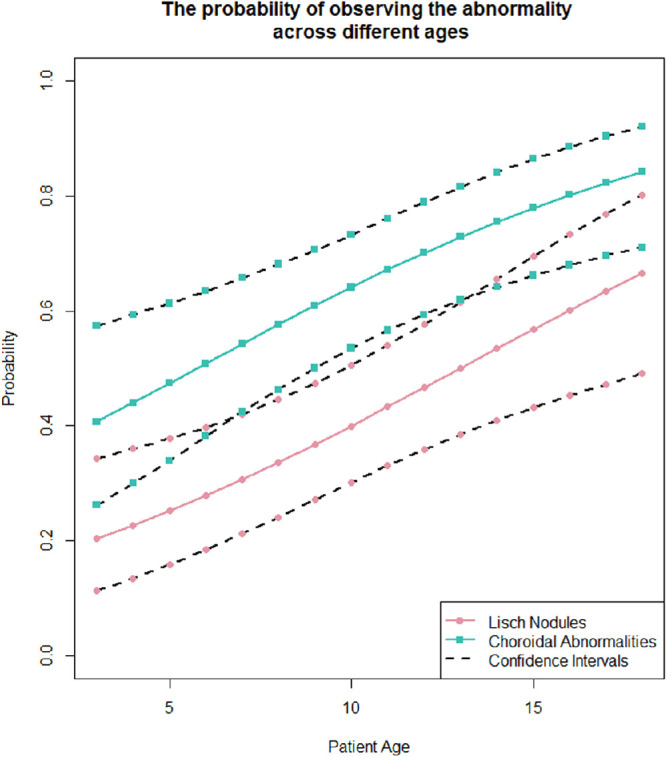
Probability of CAs and LNs observed across age groups. The *pink line* represents LNs, and the *green line* represents CAs. The confidence intervals are shown in interrupted *dashed black lines*.

**Figure 2.**
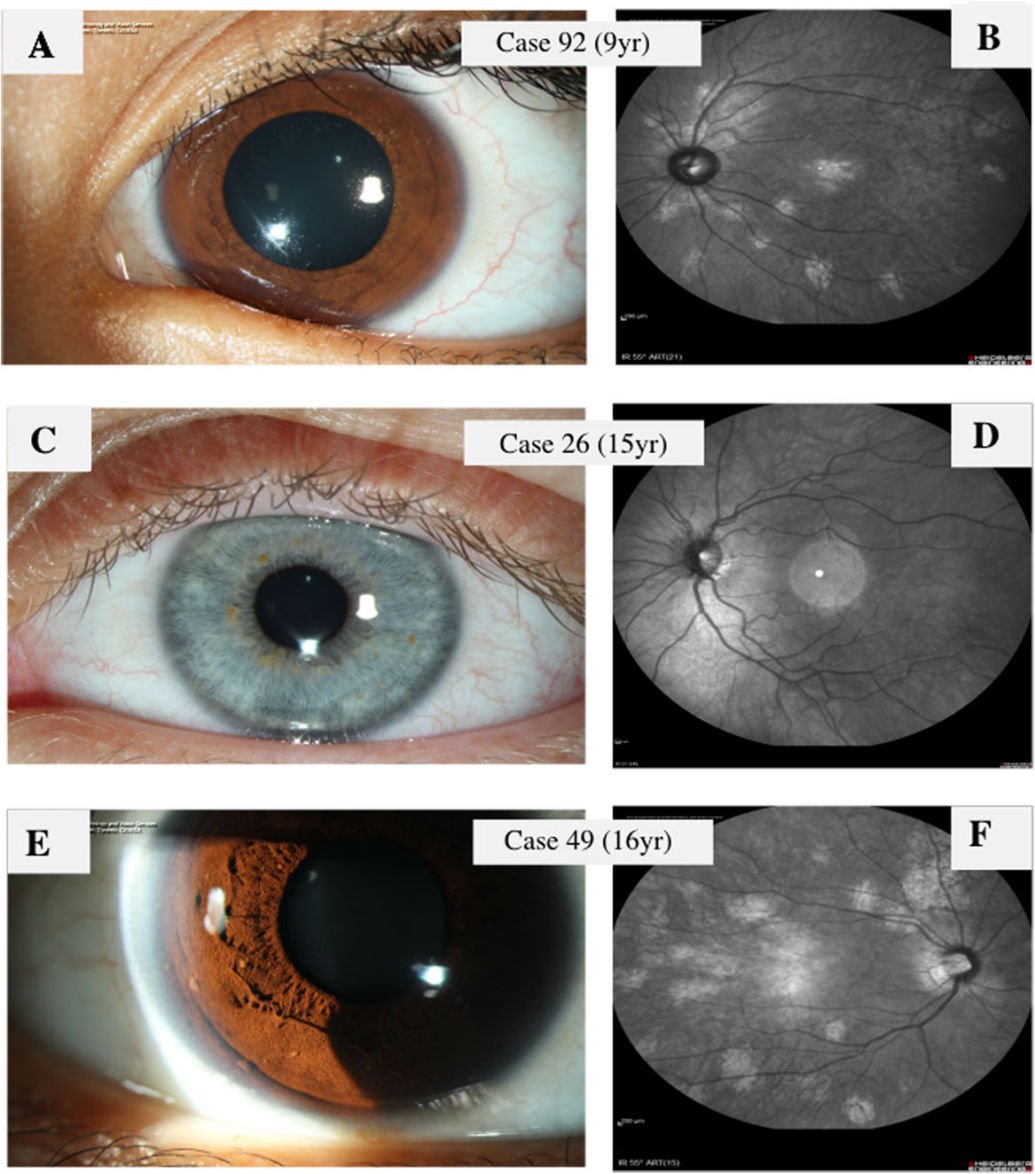
Three representative cases. (**A, B**) Case 92, a 9-year-old child with MCD who had CAs but no LNs. (**C, D**) Case 26, a 15-year-old with CDC who had LNs only. (**E, F**) Case 49, a 16-year-old with MCD who had both LNs and CAs.

**Figure 3.**
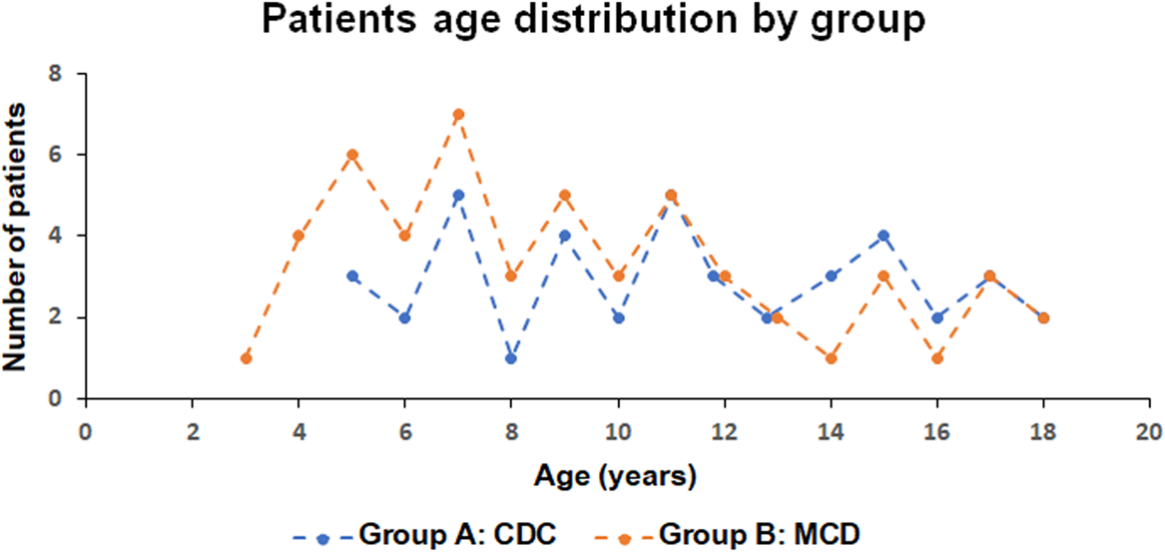
Scatterplot showing age distribution in the two subgroups. Group A patients satisfied the CDC, and group B patients additionally had a MCD. The age distribution was similar in the two groups.

All patients underwent slit-lamp examinations and NIR imaging (74 on the SPECTRALIS and 20 on the Cirrus); 34 patients (36%) additionally had anterior segment photos. The proportion of patients with CAs was 64% (95% CI, 0.53–0.73) versus patients with LNs, which was 41% (95% CI, 0.32–0.52). This difference was statistically significant: 0.22 (95% CI, 0.082–0.36; *P* = 0.0023). The probability of LNs was lower than that for CAs across all ages (odds ratio = 0.37; 95% CI, 0.20–0.69; *P* = 0.00173), with the predictive intervals overlapping for younger and older patients (shown as dashed lines in Fig. 1). In this entire group, 37% presented with CAs only, whereas 16% of the patients had LNs only. Further, 79.79% of patients had either CAs or LNs, or both. On the other hand, 20.21% of patients had neither CAs nor LNs. Figure 2 demonstrates three case examples with CAs and/or LNs. Most patients in this cohort had a MCD (group B, *n* = 53), and 41 patients met the CDC (group A). Table 1 shows the general characteristics and prevalence of CAs and LNs in the study population. As the confidence intervals overlapped (Table 1), there was no significant difference in age between the groups (*P* = 0.15). Figure 3 shows the patient age distribution in the two groups.

**Table 1.** General Characteristics and Prevalence of Ocular Findings Among the Study Population

	Entire Cohort (*N* = 94)	Group A. CDC (*n* = 41)	Group B. MCD (*n* = 53)
Age (y), mean ± SD	10.25 ± 4.15	10.17 ± 4.08	10.24 ± 4.14
Male/female, *n* (%)	38 (40)/56 (60)	18 (44)/23 (56)	20 (38)/33 (62)
Choroidal abnormalities, *n* (%)	60 (64)	28 (68)	32 (60)
Lisch nodules, *n* (%)	39 (41)	21 (51)	18 (34)
Choroidal abnormalities + Lisch nodules, *n* (%)	25 (27)	14 (34)	11 (21)
Choroidal abnormalities or Lisch nodules or both, *n* (%)	75 (80)	35 (85)	40 (75)
Optic pathway glioma, *n* (%)	25 (27)	11 (27)	14 (26)
BCVA (logMAR ± SD), mean ± SD	0.21 ± 0.36	0.20 ± 0.36	0.22 ± 0.35

### Group A. Children Meeting Clinical Diagnostic Criteria

Among the 41 patients in this group, 23 were females (56%) and 18 were males (44%). Ages ranged from 5 to 18 years (median, 10 years; mean ± SD, 10.17 ± 4.08 years). The prevalence of CAs was higher than that of LNs, but the difference did not attain statistical significance: CA prevalence, 68% (95% CI, 0.52–0.81); LN prevalence, 51% (95% CI, 0.35–0.67); and difference, 0.17 (95% CI, −0.06 to 0.40; *P* = 0.18).

### Group B. Children with Molecularly Confirmed Diagnosis

Fifty-three patients had a confirmed molecular diagnosis (33 females, 62%; 20 males, 38%). Ages ranged from 3 to 18 years (median, 10 years; mean ± SD, 10.24 ± 4.14 years). The prevalence of CAs was higher than that of LNs, and the difference was statistically significant: CA prevalence, 60% (95% CI, 0.46–0.73); LN prevalence, 34% (95% CI, 0.22–0.48); and difference, 0.26 (95% CI, 0.0062–0.47; *P* = 0.023).

### Genetic Variants

The pathogenicity of all the variants reported across the years (2001–2020) was reanalyzed using the 2015 American College of Medical Genetics and Genomics (ACMG) guidelines.^27^^,^^28^ All but one patient (case 30) had variants classified as pathogenic or likely pathogenic. Table 2 shows all the genetic variants with their ACMG classifications. Case 30 had a synonymous variant, c.987A>G/p.(Lys329=), conserved across major vertebrates and flies (phastCons^29^: 1 [0–1], phyloP^29^: 2.38 [−14.1 to 6.4]) and predicted to activate an alternative donor site upstream to the natural donor site in exon 9 (87 = >99 [0–100] for position 987 in Splicing Sequences Finder,^30^ new donor scores 4.52 [0–12], 0.58 [0–1] for position 982 in MaxEntScan^31^ and NNSPLICE,^32^ respectively). Missense variants were most frequently identified (14 patients), followed by stop (12 patients) and frameshift (10 patients) variants. Five of the variants were novel and included two missense and two frameshift variants and one whole gene deletion (involving new genomic coordinates). Table 3 shows the different class of variants and the prevalence of CAs and LNs in each mutation class; across most mutation classes, the prevalence of CAs was higher than or similar to that of LNs.

**Table 2.** Genetic Variants and ACMG Classification Guidelines

ID	Variant Position (hg19) GRCh37	Coding Position NF1	Protein Effect	Type of Variant	Supporting Evidence	ACMG Classification
1	Chr17: g.29683544C>A	NM_001042492.2:c.7682C>A	p.(Ser2540*)	Stop mutation	PVS1, PM2, PP5	Pathogenic
4	Chr17:g.29496901C>G	NM_001042492.2:c.480-8C>G	p.(?)	Splice-site mutation	PM2, PP3, PP5, PM6	Likely pathogenic
8	Chr17:g.29490327G>C	NM_001042492.2:c.412G>C	p.(Ala138Pro)	Missense	PM2, PM1, PP2, PP3, PP5	Likely pathogenic
9	Chr17:g.29585447del	NM_001042492.2:c.4259del	p.(Leu1420Tyrfs*8)	Frameshift	PVS1, PM2	Likely pathogenic
10	Chr17:g.29490327G>C	NM_001042492.2:c.412G>C	p.(Ala138Pro)	Missense	PM2, PM1, PP2, PP3, PP5	Likely Pathogenic
13	Chr17:g.29422055_29701173del		p.(?)	Whole gene deletion	1A, 2A–2E, 2H, 3A, 4L	Pathogenic
15^a^	Chr17:g.29670123_29670128del	NM_000267.3:c.7096_7101del	p.(Asn2366_Phe2367del)	Deletion, in frame	PP1, PM2, PM4, PM1, PP5	Pathogenic
21	Chr17g:25248166-30645676		p.(?)	Whole gene deletion	1A, 2A–2E, 2H, 3C, 4L	Pathogenic
22	Chr17:g.29533378C>T	NM_001042492.2:c.1381C>T	p.(Arg461*)	Stop mutation	PVS1, PM2, PP5, PM6	Pathogenic
23	Chr17:g.29556079C>T	NM_001042492.2:c.2446C>T	p.(Arg816*)	Stop mutation	PVS1, PM2, PP5, PS2, PP1	Pathogenic
25	Chr17:g.29585518A>C	NM_000267.3:c.4267A>C	p.(Lys1423Gln)	Missense	PM1, PP2, PM2, PM5, PP3, PP5, PM6	Pathogenic
27	Chr17:g.29557401G>A	NM_001042492.2:c.3113+1G>A	p.(?)	Splice-site mutation	PVS1, PM2, PP5, PM6	Pathogenic
28	Chr17:g.29422388G>C	NM_001042492.2:c.60+1G>C	p.(?)	Splice-site mutation	PVS1, PM2, PP5, PM6	Pathogenic
29	Chr17:g.29509582A>G	NM_001042492.2:c.787A>G	p.(Lys263Glu)	Missense	PM2, PP3, PP2, PP1, PS2	Likely pathogenic
30	Chr17:g.29527538A>G	NM_001042492.2:c.987A>G	p.(Lys329=)	Splice-site mutation	PM2, BP7	VUS
35	Chr17:g.29665756dup	NM_000267.3:c.6791dup	p.(Tyr2264*)	Frameshift	PVS1, PM2, PP5, PS2, PP1	Pathogenic
36	Chr17:g.29422055_29701173del		p.(?)	Whole gene deletion	1A, 2A–2E, 2H, 3A, 4L	Pathogenic
38	Chr17:g.29553638dup	NM_001042492.2:c.2187dup	p.(Asn730*)	Stop mutation	PVS1, PM2, PP5, PM6	Pathogenic
39	Chr17:g.29585422A>G	NM_000267.3:c.4171A>G	p.(Arg1391Gly)	Missense	PM1, PP2, PM2, PM5, PP3, PP5, PS2, PP1	Pathogenic
40	Chr17:g.29585518A>G	NM_001042492.2:c.4330A>G	p.(Lys1444Glu)	Missense, splicing	PM1, PP2, PM2, PM5, PP3, PP5	Pathogenic
44	Chr17:g.29684004C>T	NM_000267.3:c.7702C>T	p.(Gln2568*)	Stop mutation	PVS1, PM2, PP5, PM6	Pathogenic
45	Chr17:g.29560048_29560049del	NM_001042492.2:c.3525_3526del	p.(Arg1176Serfs*18)	Frameshift	PVS1, PM2, PP5, PS2, PP1	Pathogenic
46^a^	Chr17:g.29670123_29670128del	NM_000267.3:c.7096_7101del	p.(Asn2366_Phe2367del)	Deletion, in frame	PM2, PM4, PM1, PP5	Pathogenic
48	Chr17:g.29653042_29653043del	NM_000267.3:c.4977_4978del	p.(Tyr1659*)	Stop mutation	PVS1, PM2, PP1	Pathogenic
49	Chr17:g.29685586_29685587dup	NM_000267.3:c.7996_7997dup	p.(Ser2666Argfs*53)	Frameshift	PVS1, PM2, PM6	Pathogenic
51	Chr17:g.29541542A>G	NM_001042492.2:c.1466A>G	p.(Tyr489Cys)	Missense	PM2, PM1, PP2, PP5, PS2, PP1	Pathogenic
52	Chr17:g.29554291del	NM_001042492.2:c.2307del	p.(Thr770Leufs*21)	Frameshift	PVS1, PM2, PP1	Pathogenic
53	Chr17:g.29576004del	NM_001042492.2:c.3977del	p.(Leu1326*)	Stop mutation	PVS1, PM2, PM6	Pathogenic
55	Chr17:g.29527570_29527571del	NM_001042492.2:c.1019_1020del	p.(Ser340Cysfs*12)	Frameshift	PVS1, PM2, PP5, PM6	Pathogenic
59	Chr17:g.29528489C>T	NM_001042492.2:c.1246C>T	p.(Arg416*)	Stop mutation	PVS1, PM2, PP5, PM6	Pathogenic
61	Chr17:g.29653271G>T	NM_000267.3:c.5205+1G>T	p.(?)	Splice-site mutation	PVS1, PM2, PP5, PM6	Pathogenic
62	Chr17:g.29548947G>A	NM_001042492.2:c.1721G>A	p.(Ser574Asn)	Missense, splicing	PM2, PM5, PP2, PP5, PM6	Likely pathogenic
67	Chr17:g.29585383C>T	NM_000267.3:c.4132C>T	p.(Gln1378*)	Stop mutation	PVS1, PM2, PP5, PM6	Pathogenic
68	Chr17:g.29665757C>A	NM_000267.3:c.6792C>A	p.(Tyr2264*)	Stop mutation	PVS1, PM2, PP5	Pathogenic
69	Chr17:g.29586049G>C	NM_000267.3:c.4270-1G>C	p.(?)	Splice-site mutation	PVS1, PM2, PP5, PM6	Pathogenic
72	Chr17:g.29422055_29701173del		p.(?)	Whole gene deletion	1A, 2A–2E, 2H, 3A, 4L	Pathogenic
73^a^	Chr17:g.29533378C>T	NM_001042492.2:c.1381C>T	p.(Arg461*)	Stop mutation	PVS1, PM2, PP5	Pathogenic
74^a^	Chr17:g.29533378C>T	NM_001042492.2:c.1381C>T	p.(Arg461*)	Stop mutation	PVS1, PM2, PP5	Pathogenic
75	Chr17:g.29585403_29701083del	NM_000267.3:c.4152_8367del	p.(Gly1385Aspfs*41)	Multiple exon deletion	2A–2E, 4L	Pathogenic
76	Chr17:g.29556250C>G	NM_001042492.2:c.2617C>G	p.(Arg873Gly)	Missense	PM2, PP2, PM6, PS3	Likely pathogenic
78	Chr17:g.29508766_29508767del	NM_001042492.2:c.693_694del	p.(Thr232Lysfs*9)	Frameshift	PVS1, PM2, PS2, PP1	Pathogenic
79	Chr17:g.29559190dup	NM_001042492.2:c.3297dup	p.(Ser1100Ilefs*6)	Frameshift	PVS1, PM2, PP1	Pathogenic
80	Chr17:g.29497017T>C;	NM_001042492.2:c.586+2T>C	p.(?)	Splice-site mutation	PVS1, PM2, PP5	Pathogenic
82	Chr17:g.?	NM_001042492.2:?	p.(Gln97Valfs*13)	Exon 4 deletion	PVS1, PS1, PS2, PM2	Pathogenic
83	Chr17:g.29560162_29560164del	NM_001042492.2:c.3639_3641del	p.(Met1215del)	Deletion, in frame	PM2, PM4, PM1, PP5, PS2, PP1	Pathogenic
84	Chr17:g.29557331T>G	NM_001042492.2:c.3044T>G	p.(Leu1015Arg)	Missense	PM2, PM1, PP2, PP1	Likely pathogenic
85	Chr17:g.29541542A>G	NM_001042492.2:c.1466A>G	p.(Tyr489Cys)	Missense, splicing	PM2, PM1, PP2, PP5, PM6	Pathogenic
86	Chr17:g.29654862G>T	NM_000267.3:c.5546+5G>T	p.(?)	Splice-site mutation	PM2, PP3, PP5	Likely pathogenic
88	Chr17:g.29654736C>A	NM_000267.3:c.5425C>A	p.(Arg1809Ser)	Missense	PM2, PM1, PP2, PP3, PP5	Likely pathogenic
89	Chr17:g.29556173T>C	NM_001042492.2:c.2540T>C	p.(Leu847Pro)	Missense	PM1, PP2, PM2, PP5, PS2, PP1	Pathogenic
91	Chr17:g.29563030del	NM_001042492.2:c.3965del	p.(Asp1322Valfs*5)	Frameshift	PSV1, PM2, PP5, PM6	Pathogenic
92	Chr17:g.29676190_29676193del	NM_000267.3:c.7179_7182del	p.(Thr2394Tyrfs*2)	Frameshift	PVS1, PM2, PP5, PM6	Pathogenic
93	Chr17:g.29586057A>C	NM_000267.3:c.4277A>C	p.(Gln1426Pro)	Missense	PM1, PP2, PM2, PP3, PP5, PS2, PP1	Pathogenic

a Families with siblings.

**Table 3.** Molecular Variants and Prevalence of CAs and LNs

Patients, *n*	Type of Variant	Age (y), Range	CA Prevalence, % (*n*)	LN Prevalence, % (*n*)
14	Missense	3–17	64 (9)	29 (4)
12	Stop mutation	5–18	67 (8)	25 (3)
10	Frameshift	5–16	60 (6)	50 (5)
8	Splice-site mutation	4–12	62 (5)	12 (1)
4	Whole gene deletion	5–17	25 (1)	50 (2)
3	In-frame deletion	13–17	100 (3)	100 (3)
2	Exon deletion	4–5	0 (0)	50 (1)

### Comparison Between Group A and Group B

There was no statistically significant difference in the prevalence of CAs among the patients with a clinical (68%) or molecularly confirmed (60%) diagnosis (−0.079; 95% CI, −0.30 to 0.13). Although a larger proportion of those in group A were found to have LNs (51% vs. 34%), this difference was not statistically significant (−0.17; 95% CI, −0.39 to 0.05).

## Discussion

This study is the largest pediatric cohort to describe CAs and its relationship with LNs in molecular confirmed neurofibromatosis 1; CAs were found to be more prevalent than LNs in our genetically confirmed cohort. Further, these CAs were more prevalent than LNs across all age groups regardless of their diagnosis status (CDC or MCD). Nearly 80% of children in this study met the latest NF1 diagnostic guidelines, which added CAs in addition to LNs,^4^ counting for one point toward the diagnostic criteria. Moreover, in this cohort, there was a trend for CAs to present earlier than LNs, a finding not reported previously, to the best of our knowledge.

### Prevalence of CAs Is Higher Compared to LNs

In the current study (entire cohort), CAs were found more frequently in patients with NF1 than were LNs (64% vs. 41%). The prevalence of CAs was similar between the two subgroups (68% in CDC and 60% in MCD), but the prevalence of LNs was more variable between the CDC and MCD subgroups (51% and 34%, respectively). The variability in the prevalence of LNs may be attributed to the variable expressivity of LNs in NF1^7^^–^^9^ and to the subjectivity of slit-lamp examinations (as reported in the literature).^16^ The reported prevalence of CAs in the pediatric NF1 literature has ranged from 60.5% to 78.9%,^7^^,^^8^^,^^12^^,^^15^ higher than the prevalence of LNs (43%–62%),^7^^,^^8^^,^^12^^,^^15^ similar to our results. However, a few study results are different.^8^^,^^33^ Parrozzani et al.^8^ reported a similar prevalence for CAs (60.5%; *n* = 129) and LNs (62.5%; *n* = 119). At the end of a 6-year prospective study of predominantly pediatric cohort, Chilibeck et al.^33^ reported a lower prevalence of CAs (70%) than LNs (80%); further, at the final exam, patients ≤ 5 years of age had LNs more frequently than CAs (28.8% vs. 15%).

### CAs Appear Earlier Than LNs

In the current study, the presence of CAs and LNs increased with age, as previously described.^7^^,^^18^^–^^21^ This highlights the importance of examining for both CAs and LNs in NF1 patients. Further, in our cohort, there was a trend for CAs to present earlier than LNs, a novel finding not reported in the literature. In this cohort, the youngest patient who demonstrated CAs was 4 years old, whereas the youngest with LNs was 5 yeas old.

### CAs Found in Significant Proportion of Pediatric NF1 Patients Without LNs

In the present study, 37% (*n* = 35) of the patients had CAs only, which was similar among the CDC (34%; *n* = 14) and MCD (39%; *n* = 21) subgroups. This occurrence is higher than reported by Vagge et al.,^12^ Viola et al.,^7^ and Parrozzani et al.,^8^ who reported 24%, 21%, and 18%, respectively, and is much higher than what was reported by Chilibeck et al.^33^ (5%). Given the subjectivity and lower occurrence of LNs in children, the presence of CAs exclusively in one-fifth to one-third of pediatric patients highlights the diagnostic value of NIR in NF1. Further, Parrozzani et al.^8^ concluded that the presence of CAs had a positive predictive value of 0.98 and a negative predictive value of 0.46 in children with a clinical diagnosis of NF1.

### LNs Found in Lower Proportion of Pediatric NF1 Patients Without CAs

In the current study, 16% (*n* = 15) of the patients had LNs only, which was similar between the CDC (17%; *n* = 7) and MCD (15%; *n* = 8) subgroups. This occurrence was similar to the findings of Chilibeck et al.^33^ (15%) and Parrozzani et al.^8^ (19%) but higher compared to Viola et al.^7^ (9%) and Vagge et al.^12^ (4%). Chilibeck et al.^33^ reported that those patients who did not have CAs in their first examination did not develop any over the course of the 6-year study period. Taken together, examining patients with suspected NF1 for LNs continues to be of diagnostic importance.

### Inclusion of CAs in NF1 Criteria Increases Diagnostic Reach of Ophthalmic Manifestations

In our entire cohort, 26.6% (*n* = 25) showed both LNs and CAs; our results differ from those of other pediatric studies, which have reported a higher concurrent occurrence of both ocular signs (42.86%–65%).^8^ However, 79.79% (*n* = 75) of our patients presented with one of the signs (CAs or LNs) or both, similar to reports in the literature ranging from 74.42% to 92.63%.^7^^,^^8^^,^^12^^,^^15^ Further, 37% (*n* = 35) of our patients had CAs alone. The recent diagnostic criteria for NF1 assign a criteria point for the presence of either two CAs or two LNs, whereas previous criteria did not include CAs.^2^^,^^4^ Our results further support that the inclusion of CAs in the diagnostic criteria will increase the diagnostic reach of ocular findings in patients with suspected NF1. Although the presence of a bright choroidal patch mimicking CAs has been reported in two cases of Legius syndrome, neither of those cases had more than one lesion^18^; hence, the diagnostic cutoff of two CAs for NF1 seems adequate.

### Limitation of CAs and LNs in Diagnosis of NF1

In our cohort, 20.21% (*n* = 19) had neither CAs nor LNs; in the literature, this value has ranged from 16.25% to 26.58%,^7^^,^^8^^,^^12^^,^^15^ with one outlier reported at 7.37%.^7^ This is a key reminder that about one-fifth of pediatric patients may not present with these ocular signs.

### Genetic Variants Identified in Patients with CAs or LNs

In group B, a disease-causing variant was found in all 53 patients. Next-generation sequencing techniques have increased the molecular detection rate for NF1 diagnosis to 95% to 97%.^4^ Inactivating (loss of function) variants are described in >80% of those who meet NIH clinical criteria.^34^ Our study results are similar; missense variants were identified in 26.4% of patients, whereas severe mutations (stop, frameshift, whole gene deletion, exon deletion, in-frame deletion, and splice-site) were identified in the majority (73.6%). Further, CAs appeared to be more common in most mutation classes; however, the number of patients in each category was too low for statistical analysis to be conducted.

## Conclusions

The prevalence of CAs was similar between pediatric patients with MCD and those with clinical diagnosis only. The prevalence of CAs was significantly higher than that of LNs in the same population regardless of age; CAs tended to present at an earlier age. Further, the presence of either CAs or LNs, or both, was found in nearly 80% of the patients with NF1, highlighting the diagnostic utility of an eye exam in children with NF1. Future studies may ascertain if CAs are noticeable during infancy or early childhood (<3 years).

## References

[1] Abdolrahimzadeh B, Piraino DC, Albanese G, Cruciani F, Rahimi S. Neurofibromatosis: an update of ophthalmic characteristics and applications of optical coherence tomography. *Clin Ophthalmol*. 2016; 10: 851–860. 27257370 10.2147/OPTH.S102830 PMC4874640

[2] Office of Medical Applications of Research, National Institutes of Health. Neurofibromatosis. Conference statement. National Institutes of Health Consensus Development Conference. *Arch Neurol*. 1988; 45: 575–578. 3128965

[3] Ferner RE, Huson SM, Thomas N, et al. Guidelines for the diagnosis and management of individuals with neurofibromatosis 1. *J Med Genet*. 2007; 44: 81–88. 17105749 10.1136/jmg.2006.045906 PMC2598063

[4] Legius E, Messiaen L, Wolkenstein P, et al. Revised diagnostic criteria for neurofibromatosis type 1 and Legius syndrome: an international consensus recommendation. *Genet Med*. 2021; 23: 1506–1513. 34012067 10.1038/s41436-021-01170-5 PMC8354850

[5] Cnossen MH, de Goede-Bolder A, van den Broek KM, et al. A prospective 10 year follow up study of patients with neurofibromatosis type 1. *Arch Dis Child*. 1998; 78: 408–412. 9659085 10.1136/adc.78.5.408 PMC1717584

[6] Tonsgard JH. Clinical manifestations and management of neurofibromatosis type 1. *Semin Pediatr Neurol*. 2006; 13: 2–7. 16818170 10.1016/j.spen.2006.01.005

[7] Viola F, Villani E, Natacci F, et al. Choroidal abnormalities detected by near-infrared reflectance imaging as a new diagnostic criterion for neurofibromatosis 1. *Ophthalmology*. 2012; 119: 369–375. 21963267 10.1016/j.ophtha.2011.07.046

[8] Parrozzani R, Clementi M, Frizziero L, et al. In vivo detection of choroidal abnormalities related to NF1: feasibility and comparison with standard NIH diagnostic criteria in pediatric patients. *Invest Ophthalmol Vis Sci*. 2015; 56: 6036–6042. 26393470 10.1167/iovs.14-16053

[9] Ragge NK, Falk RE, Cohen WE, Murphree AL. Images of Lisch nodules across the spectrum. *Eye (Lond)*. 1993; 7: 95–101. 8325432 10.1038/eye.1993.20

[10] Wolter JR, Gonzales-Sirit R, Mankin WJ. Neuro-fibromatosis of the choroid. *Am J Ophthalmol*. 1962; 54: 217–225. 14040313

[11] Yasunari T, Shiraki K, Hattori H, Miki T. Frequency of choroidal abnormalities in neurofibromatosis type 1. *Lancet*. 2000; 356: 988–992. 11041400 10.1016/S0140-6736(00)02716-1

[12] Vagge A, Camicione P, Capris C, et al. Choroidal abnormalities in neurofibromatosis type 1 detected by near-infrared reflectance imaging in paediatric population. *Acta Ophthalmol*. 2015; 93: e667–e671. 25990002 10.1111/aos.12750

[13] Moramarco A, Giustini S, Nofroni I, et al. Near-infrared imaging: an in vivo, non-invasive diagnostic tool in neurofibromatosis type 1. *Graefes Arch Clin Exp Ophthalmol*. 2018; 256: 307–311. 29290016 10.1007/s00417-017-3870-z

[14] Abdolrahimzadeh S, Felli L, Plateroti R, et al. Morphologic and vasculature features of the choroid and associated choroid-retinal thickness alterations in neurofibromatosis type 1. *Br J Ophthalmol*. 2015; 99: 789–793. 25488947 10.1136/bjophthalmol-2014-306062

[15] Goktas S, Sakarya Y, Ozcimen M, et al. Frequency of choroidal abnormalities in pediatric patients with neurofibromatosis type 1. *J Pediatr Ophthalmol Strabismus*. 2014; 51: 204–208. 24844395 10.3928/01913913-20140513-02

[16] Beauchamp GR. Neurofibromatosis type 1 in children. *Trans Am Ophthalmol Soc*. 1995; 93: 445–472. 8719691 PMC1312070

[17] Nakakura S, Shiraki K, Yasunari T, Hayashi Y, Ataka S, Kohno T. Quantification and anatomic distribution of choroidal abnormalities in patients with type I neurofibromatosis. *Graefes Arch Clin Exp Ophthalmol*. 2005; 243: 980–984. 15891894 10.1007/s00417-005-1184-z

[18] Cassiman C, Casteels I, Jacob J, et al. Choroidal abnormalities in cafe-au-lait syndromes: a new differential diagnostic tool? *Clin Genet*. 2017; 91: 529–535. 27716896 10.1111/cge.12873

[19] Rao RC, Choudhry N. Enhanced depth imaging spectral-domain optical coherence tomography findings in choroidal neurofibromatosis. *Ophthalmic Surg Lasers Imaging Retina*. 2014; 45: 466–468. 25153660 10.3928/23258160-20140818-01 PMC4398345

[20] Kumar V, Singh S. Multimodal imaging of choroidal nodules in neurofibromatosis type-1. *Indian J Ophthalmol*. 2018; 66: 586–588. 29582830 10.4103/ijo.IJO_1095_17 PMC5892072

[21] Ishiko S, Yoshida A, Kato Y, Kagokawa H. Occult retinal and choroidal lesions in neurofibromatosis type 1. *Br J Ophthalmol*. 2006; 90: 1067–1068. 10.1136/bjo.2006.092718 PMC1857214 16854838

[22] McAnany JJ, Genead MA, Walia S, et al. Visual acuity changes in patients with Leber congenital amaurosis and mutations in CEP290. *JAMA Ophthalmol*. 2013; 131: 178–182. 23411883 10.1001/2013.jamaophthalmol.354 PMC3688627

[23] Makino S, Tampo H, Arai Y, Obata H. Correlations between choroidal abnormalities, Lisch nodules, and age in patients with neurofibromatosis type 1. *Clin Ophthalmol*. 2014; 8: 165–168. 24403820 10.2147/OPTH.S56327 PMC3883548

[24] R Foundation. *The R Project for Statistical Computing*. Available at: https://www.r-project.org/ . Accessed January 21, 2022.

[25] Hosmer DW Jr, Lemeshow S, Sturdivant RX. *Applied logistic regression*. 3rd ed. Hoboken, NJ: John Wiley & Sons; 2013.

[26] Benjamini Y, Hochberg Y. Controlling the false discovery rate: a practical and powerful approach to multiple testing. *J R Stat Soc Ser B Methodol*. 1995; 57: 289–300.

[27] Richards S, Aziz N, Bale S, et al. Standards and guidelines for the interpretation of sequence variants: a joint consensus recommendation of the American College of Medical Genetics and Genomics and the Association for Molecular Pathology. *Genet Med*. 2015; 17: 405–424. 25741868 10.1038/gim.2015.30 PMC4544753

[28] Brandt T, Sack LM, Arjona D, et al. Adapting ACMG/AMP sequence variant classification guidelines for single-gene copy number variants. *Genet Med*. 2020; 22: 336–344. 31534211 10.1038/s41436-019-0655-2

[29] Siepel A, Bejerano G, Pedersen JS, et al. Evolutionarily conserved elements in vertebrate, insect, worm, and yeast genomes. *Genome Res*. 2005; 15: 1034–1050. 16024819 10.1101/gr.3715005 PMC1182216

[30] Pollard KS, Hubisz MJ, Rosenbloom KR, Siepel A. Detection of nonneutral substitution rates on mammalian phylogenies. *Genome Res*. 2010; 20: 110–121. 19858363 10.1101/gr.097857.109 PMC2798823

[31] Desmet FO, Hamroun D, Lalande M, Collod-Béroud G, Claustres M, Béroud C. Human Splicing Finder: an online bioinformatics tool to predict splicing signals. *Nucleic Acids Res*. 2009; 37: e67. 19339519 10.1093/nar/gkp215 PMC2685110

[32] Eng L, Coutinho G, Nahas S, et al. Nonclassical splicing mutations in the coding and noncoding regions of the ATM gene: maximum entropy estimates of splice junction strengths. *Hum Mutat*. 2004; 23: 67–76. 14695534 10.1002/humu.10295

[33] Chilibeck CM, Shah S, Russell HC, Vincent AL. The presence and progression of choroidal neurofibromas in a predominantly pediatric population with neurofibromatosis type-1. *Ophthalmic Genet*. 2021; 42: 223–229. 33594930 10.1080/13816810.2021.1881977

[34] Philpott C, Tovell H, Frayling IM, Cooper DN, Upadhyaya M. The NF1 somatic mutational landscape in sporadic human cancers. *Hum Genomics*. 2017; 11: 13. 28637487 10.1186/s40246-017-0109-3 PMC5480124

